# The barriers and facilitators to managing diabetes with insulin in adults with intellectual disabilities: A systemised review of the literature

**DOI:** 10.1111/jar.13027

**Published:** 2022-08-18

**Authors:** Cathy J. Beresford, Olga Kozlowska

**Affiliations:** ^1^ Diabetes Centre King Edward VII Hospital Windsor Berkshire UK; ^2^ Oxford Brookes University Oxford UK

**Keywords:** diabetes, insulin, intellectual disabilities, reasonable adjustments, self‐management, support

## Abstract

**Background:**

People with intellectual disabilities are more likely to have diabetes and develop complications from it. Diabetes management is complex and insulin treatment in particular, people with intellectual disabilities may require additional support that is not always available. This review aimed to identify barriers and facilitators to managing diabetes with insulin in adults with intellectual disabilities.

**Method:**

Patient and public involvement (PPI) was integral to the development of the research question. A systemised review was conducted across CINAHL, the British Nursing Index and MEDLINE. PRISMA guidelines were followed. Narrative synthesis of the evidence was undertaken.

**Results:**

Barriers and facilitators to managing diabetes with insulin in people with intellectual disabilities were identified related to the individual, other people participating in their care, and broader environmental and social factors.

**Conclusions:**

People with intellectual disabilities who use insulin, require reasonable adjustments to education, support, and a person‐centred approach to facilitate supported self‐management. More training for their supporters is needed and further inclusive research with PPI is recommended.

## 1 INTRODUCTION

In the United Kingdom, in 2019, there were 3.9 million people identified as having diabetes and it is estimated that a further million people have it, but have not been diagnosed yet (Diabetes UK, 2020). According to the World Health Organisation, 422 million people worldwide have diabetes, with numbers estimated to rise to over half a billion by 2030 (WHO, 2021). Diabetes is characterised by raised blood glucose levels (hyperglycaemia) resulting from partial or total insufficiency in the insulin hormone (Egan & Dinneen, 2019). This serious long‐term condition requires management to prevent health complications including cardiovascular disease, kidney disease, neuropathy, problems with the eyes and feet (Egan & Dinneen, 2019).

Compared with the general population, diabetes affects people with intellectual disabilities disproportionately, they have 2.46 times higher odds for developing diabetes, with 8.5% of people with intellectual disabilities having the condition (Vancampfort et al., 2022). People with intellectual disabilities experience poorer health outcomes (Cooper et al., 2018) and are at increased risk of developing diabetes complications due to healthcare inequalities (Hanlon et al., 2018; MacRae et al., 2015). There are higher rates of hospitalisation among people with intellectual disabilities, with evidence of barriers in accessing diabetes screening and health checks within primary care (Dunn et al., 2018; Hanlon et al., 2018). The World Health Organisation (WHO, 2012) identifies intellectual disabilities as an area for research priority and the NHS (2019) long‐term plan includes improving understanding of the needs of people with disabilities and reducing inequalities in health. Issues of health inequality for people with intellectual disabilities have been further amplified by the COVID‐19 pandemic (Williamson et al., 2021).

There are two main types of diabetes, both of which are more prevalent in people with intellectual disabilities (NHS Digital, 2019): type 1 accounts for about 8% of cases and type 2 approximately 90% (Diabetes UK, 2020). Insulin therapy is required for all people with type 1 and some with type 2 (Diabetes UK, 2020) and can be complex because it requires blood glucose monitoring, self‐injections or insulin pump, dose adjustment, dietary considerations, and management of high or low blood glucose. There are safety issues around insulin therapy, placing it in the top high‐alert medicines internationally (Cousins et al., 2011).

There is growing awareness of the importance of making reasonable adjustments to support people with intellectual disabilities to manage their diabetes (NHS Rightcare, 2017) and an increase in research focussing on these matters. However, some issues, including barriers and facilitators to diabetes management with insulin, need more attention because people with intellectual disabilities experience inequitable access to diabetes education and healthcare (Brown et al., 2017; Smith & Phillips, 2018). Better understanding of these will help in developing interventions to support people with intellectual diabetes and diabetes and therefore improve their health outcomes.

## 2 METHODS

### 2.1 Review design

A systemised review design was used, with the review process following the principles of a systematic review but limited to published peer‐reviewed academic literature and a narrative synthesis of findings (Grant & Booth, 2009). The Preferred Reporting Items for Systematic Reviews and Meta‐Analyses (PRISMA) guidelines were followed (Moher et al., 2009). The review protocol was written as a summative assignment by the lead reviewer as part of a module in Applied Research Methods at Oxford Brookes University.

### 2.2 Patient and public involvement (PPI)

PPI was integral to developing the research question for this literature review (Brand et al., 2020). The lead author is a Senior Diabetes Specialist Nurse in an outpatient department and facilitated a PPI meeting in 2019 attended by people with intellectual disabilities and their supporters. The group discussed living with intellectual disabilities and diabetes. It was agreed that diabetes is a challenging condition to manage and even more so for people with intellectual disabilities using insulin. The group were strongly in favour of a research project to focus on this:

‘…help people out and find out new ways of doing this’ (person with intellectual disability and diabetes).

‘…a very much needed line of research for an area that doesn't receive enough attention’ (sister of a person with Down's syndrome and diabetes).

‘People with learning disabilities need a voice and help to manage their diabetes’ (person with intellectual disability and diabetes).

### 2.3 Developing the research question

An initial probe of the literature found that diabetes management in people with intellectual disabilities has been increasingly researched in recent years, but there is a lack of focus on how those using insulin manage their diabetes. Informed with the feedback from the PPI meeting, the research question was formed using the PEO question format (Munn et al., 2018); *in adults with diabetes* (population) *and intellectual disabilities* (exposure) *what are the barriers and facilitators to managing diabetes with insulin* (outcome)?

### 2.4 Search strategy

There was consensus between the reviewers to search the following databases: Cumulative Index of Nursing and Allied Health Literature (CINAHL), the British Nursing Index and MEDLINE, as these were considered most relevant to the topic. The searches were conducted by the lead reviewer between December 2021 and January 2022. Support was sought from the university health librarian to ensure a robust search using appropriate key terms (Table 1). Reference lists of retrieved papers were checked manually by the lead reviewer.

**TABLE 1:** Search terms included

Keyword	Learning disab* or Intellectual disab* or Cognitive disab* or Down syndrome
And	Diabet*
And	Insulin or Inject* or Medic* or Self manag* or glucose test* or glucose monitor*

### 2.5 Selection criteria

Inclusion criteria:

- Peer‐reviewed publications of primary research related to adults with an intellectual disability and diabetes (with no date restriction for publications)
- Mention of medication/injections/insulin/blood glucose monitoring (in this population)
- Mention of barriers/facilitators to diabetes management (in this population)
- Published in English

Exclusion criteria:

- Publications focusing on diabetes in people *without* intellectual disability
- Participants <18 years. Studies about people <18 were excluded because diabetes services for children are provided separately to adult services and children are offered a different level of support to adults
- No reference to medication or blood glucose testing
- No barriers or facilitators *to managing diabetes with insulin* identified
- Non‐peer reviewed journal articles

### 2.6 Screening process

**FIGURE 1:**
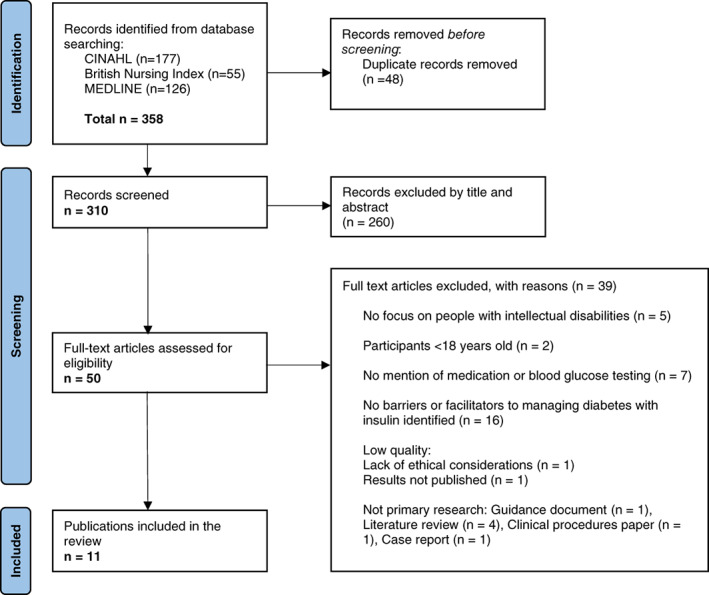
PRISMA flowchart. *Source*: Page et al. (2021)

Figure 1 shows the PRISMA flowchart (Page et al., 2021). A total of 358 papers were exported to EndNote. Duplicates were removed (*n* = 48) and there were 310 records assessed against the selection criteria via title and abstract screening (*n* = 260 excluded). This left 50 records to be assessed for eligibility. Full‐text articles excluded for not meeting selection criteria *n* = 29. A total of 21 full text papers were appraised. During the appraisal process two papers were excluded based on low quality – one had no mention of ethical considerations, including consent, and the other did not include the results of the study. Two papers were excluded because they were not primary research, one paper was excluded because it was specific to people with cognitive impairment, such as dementia, rather than intellectual disability. Four papers were excluded as they were literature reviews. One paper was excluded as it was a guidance document. Among included studies, a published and peer‐reviewed service improvement project was included (Gregory, 2019) because it reported specifically on a project to improve service standards for people who are housebound requiring insulin. A total of 11 papers were included in this literature review, see Table 2.

**TABLE 2:** Overview of the included publications

Authors (year), location of study	Title	Aim of the study	Study design participants methodology	Were the perspectives of people with intellectual disabilities collected in the research?	Quality appraisal
Brown et al. (2017), Scotland	Improving diabetes care for people with intellectual disabilities: a qualitative study exploring the perceptions and experiences of professionals in diabetes and intellectual disability services.	To explore the perceptions and experiences of health and social care practitioners caring for people with intellectual disabilities who have diabetes and identify their education needs and service development opportunities.	Qualitative study. Semi‐structured interviews with 29 professionals/support workers. Thematic analysis.	No	High
Dysch et al. (2012), UK	How do people with intellectual disabilities and diabetes experience and perceive their illness?	To utilise a qualitative approach to explore the subjective experiences and perceptions of people with intellectual disabilities and diabetes.	Qualitative study. Semi‐structured interviews with four people with intellectual disabilities and diabetes. Interpretative Phenomenological Analysis.	Yes	High
Hale et al. (2011), Canterbury, New Zealand	Self‐management abilities of diabetes in people with an intellectual disability living in New Zealand.	To examine how diabetes management is actually carried out as well as how skilfulness is perceived by the adults and then to provide information on the knowledge and understanding of diabetes held. by a select group of adults with ID.	Qualitative study. Semi‐structured interviews with 14 people with intellectual disabilities and diabetes. General inductive approach to analysis	Yes	High
Maine et al. (2017), Scotland	An application of Bandura's ‘Four Sources of Self‐Efficacy’ to the self‐management of type 2 diabetes in people with intellectual disability: An inductive and deductive thematic analysis.	To qualitatively explore the experiences of people with intellectual disabilities self‐manging type 2 diabetes, and to inductively and deductively evaluate the appropriateness of the Four Sources model for this population.	Qualitative study. Semi‐structured interviews with 10 people with intellectual disabilities and diabetes. Bandura's framework guided thematic analysis.	Yes	High
Whitehead et al. (2016), New Zealand	Negotiated autonomy in diabetes self‐management: the experiences of adults with intellectual disability and their support workers.	To explore the experience and practice of autonomy in relation to the self‐management of diabetes for those with intellectual disabilities living in residential or independent living settings and the role of their support workers.	Qualitative study. Semi‐structured interviews of 14 people with intellectual disabilities and diabetes; 17 support workers. Thematic analysis.	Yes	High
Cardol et al. (2012a), The Netherlands	Attitudes and dilemmas of caregivers supporting people with intellectual disabilities who have diabetes.	To explore how professional caregivers in communal living arrangements support people with a mild or moderate intellectual disability who have diabetes.	Qualitative study. Semi‐structured interviews of 13 professional caregivers. Grounded theory analysis.	No	Medium
Cardol et al. (2012b), The Netherlands	People with mild to moderate intellectual disability talking about their diabetes and how they manage.	To explore the diabetes perceptions of people with intellectual disabilities, examine how people manage diabetes and how they communicate with health professionals about diabetes.	Qualitative study. Semi‐structured interviews of 17 people with intellectual disabilities and diabetes. Thematic analysis.	Yes	Medium
Paterson et al. (2020), Scotland	Characteristics of diabetes medication‐taking in people with mild to moderate intellectual disability compared to those without: a mixed‐methods study.	To compare the frequency and factors associated with diabetes medication‐taking in people with mild to moderate intellectual disability and those without intellectual disability.	Two stage mixed‐methods study. Information collected on 111 people with diabetes: 33 adults with intellectual disability and 78 adults without intellectual disability. Stage 2: Qualitative semi‐structured interviews with carers. Thematic analysis.	No	Medium
Rouse and Finlay (2016), South and Midlands of England	Repertoires of responsibility for diabetes management by adults with intellectual disabilities and those who support them.	To explore repertoires of responsibility in accounts of managing diabetes for adults with intellectual disability.	Qualitative study. Semi‐structured interviews of seven adults with intellectual disability and diabetes, and seven people who they nominated as supporting their diabetes management. Critical discursive psychological approach to analysis.	Yes	Medium
Trip et al. (2016), Canterbury, New Zealand	The role of key workers in supporting people with intellectual disability in the self‐management of their diabetes: a qualitative New Zealand study.	To explore how key workers supported the self‐management of diabetes in people with intellectual disability.	Qualitative study. Semi‐structured interviews of 17 key workers (nominated by people with diabetes & intellectual disability and identified as having responsibility in facilitating primary healthcare access for the person). General Inductive Approach to thematic analysis.	No (but they are in Hale et al., 2011, which is part of the same project)	Medium
Gregory (2019), East Kent, England	Housebound patients with diabetes needing support with insulin—a project to improve service standards.	A project to improve the community nursing team's insulin administration service for vulnerable homebound adults with diabetes.	The project included training delivered by a nurse consultant to enable six healthcare assistants/associate practitioners to become competent in a basic standard in nine key diabetes competency areas – including the safe administration of insulin.	No	Low

### 2.7 Data extraction

The lead reviewer used a tailored data extraction template designed by the reviewers to capture relevant information from the included papers (see Appendix S1). The information extracted included: the country and area of the study, the study setting, the aims and objectives, the participant population, whether the perspectives of people with intellectual disabilities were included, eligibility and recruitment, the methodology and study design, data collection method and analysis, barriers, and facilitators relevant to using insulin, relevant suggestions from the authors and anything else of interest. Throughout the data extraction process, each paper was double‐checked by the second reviewer.

### 2.8 Quality assessment

The papers were assessed for quality using the adapted Critical Appraisal Skill Programme (2018) (Appendix S1); all papers were assessed by both reviewers and high level of consensus reached. Gregory (2019) was not quality assessed as a research paper because it was a service improvement project, so this was given low weighting in the data analysis process. Five papers were assessed as medium quality because there was a lack of examination of the researchers' own role and influence throughout the research process, for example, opportunity for participants to check the interview data, or discussion about the role of the researcher as a person without intellectual disability in relationship to the participants (Cardol et al., 2012a, 2012b; Paterson et al., 2020; Rouse & Finlay, 2016; Trip et al., 2016). Five papers were assessed as high quality (Brown et al., 2017; Dysch et al., 2012; Hale et al., 2011; Maine et al., 2017; Whitehead et al., 2016). To ensure the findings of the review were reliable, both reviewers conducted quality assessment and agreed on exclusion/inclusion.

The adaptation of the CASP tool was to enable consideration of additional criteria – the inclusion of Patient and Public Involvement (PPI) in the research process, and whether or not people with intellectual disabilities were participants in the research. There was little mention of PPI in the research processes of the papers or discussion about how the research questions and interview schedules were developed. An exception to this was Cardol et al. (2012b) who developed their interview protocol with a person with intellectual disabilities. It was also important to capture the extent of gathering the views of people with intellectual disabilities; this was a necessary consideration as studies exploring diabetes care without collecting perspectives of people with intellectual disabilities risk being irrelevant to them; this was done well by Cardol et al. (2012b), Dysch et al. (2012), Hale et al. (2011), Maine et al. (2017), Rouse and Finlay (2016) and Whitehead et al. (2016). Paterson et al. (2020) interviewed carers about diabetes medication‐taking but did not explain why the people with intellectual disabilities were not interviewed themselves.

### 2.9 Data analysis

The data was analysed using a systematic and rigorous coding and themes development process, aiming to combine results from included studies (Aveyard, 2019). Papers were read by both reviewers. The lead reviewer re‐read papers and made reflective notes throughout the appraisal and analysis process as recommended by Aveyard (2019). After the initial data extraction phase theme development began by focussing on the results sections of the papers with particular focus on the barriers and facilitators to managing diabetes with insulin. The papers were then re‐read with line‐by‐line analysis, to further develop the themes. Stronger studies were given more weight than weaker research, with additional consideration of whether the people with intellectual disabilities were included as participants. Thematic development was ongoing throughout the data analysis process, with the emergence of main themes and subthemes, as shown in Tables 3, 4 and 5 (Appendix S2).

## 3 RESULTS

There were three main themes, each with subthemes, related to the barriers and facilitators of managing diabetes with insulin in people with intellectual disabilities:

1. The individual factors (i.e., the person with intellectual disability and diabetes)
2. The role of other people (i.e., family/carers/healthcare professionals/support workers)
3. Social/environmental factors

Tables 6, 7 and 8 illustrate the results (see Appendix S3).

### 3.1 Theme 1: The individual factors

#### 3.1.1 The cognitive ability or competence of the person with intellectual disability

Cognitive ability was identified as a factor in how people with intellectual disabilities were able to manage their diabetes with insulin within all of the studies, except for Whitehead et al. (2016). Being unable (or perceived as unable) to check blood glucose or inject insulin was a barrier to self‐management, whereas being able (or perceived as able) to manage blood glucose testing and insulin injections was a facilitator. Cardol et al. (2012b) identified that as a barrier, this was overcome to some extent if the person was motivated to manage their diabetes. Maine et al. (2017) described tools that enabled the participants in their study on self‐management of type 2 diabetes in people with intellectual disabilities to overcome barriers caused by cognitive impairments, such as a large print glucose diary, calendars and medication boxes. All of the studies identified support from other people as crucial to overcoming difficulties.

How the person's ability was perceived by *other* people (e.g., healthcare professionals or support workers) seemed to be particularly relevant in terms of whether or not this was a barrier to their diabetes management, as illustrated by the results of Brown et al. (2017) in which healthcare professionals highlighted that the person's level of intellectual disability, comprehension and communication skills impacted on their ability to manage their treatment regimen. In relation to the type 1 diabetes education programme DAFNE a doctor stated, ‘there's no way in a million years that somebody with a learning disability could do that course’ (p. 443). In Cardol et al.'s (2012a) study, caregivers were sometimes negative about the ability of people with intellectual disabilities to learn and self‐manage their diabetes, using language such as ‘he doesn't know how insulin works. He'll never learn anything’ (p. 385). In contrast, Whitehead et al. (2016) described how support, encouragement and working together with people with intellectual disabilities can facilitate their autonomy to manage their diabetes. Similarly, Trip et al. (2016) quoted a support worker referring to a client: ‘she would be capable of doing it [insulin] if we just gave her support’ (p. 794).

#### 3.1.2 Knowledge

A lack of understanding about diabetes, or inadequate access to diabetes education, was a barrier to managing diabetes with insulin (Brown et al., 2017; Cardol et al., 2012a; Hale et al., 2011; Maine et al., 2017). Barriers to knowledge included a lack of accessible information or structured education for people with intellectual disabilities. In contrast, if a person was supported to understand their condition, why they needed insulin and had access to information, this facilitated their diabetes management according to data from all of the papers except Gregory (2019) and Paterson et al. (2020). Maine et al. (2017) found that diabetes knowledge facilitated confidence and competence in self‐management. For example, one of their participants enjoyed being expert in self‐administering his insulin and having competence in blood glucose monitoring. Cardol et al. (2012b) found a relationship between understanding of diabetes and being able to manage it better.

#### 3.1.3 Motivation/mood

This was identified as a facilitator within seven studies, and within five of the studies as a barrier. If a person with diabetes had low mood or was not motivated to do their own injections and/or check blood glucose levels, this affected their ability to manage their diabetes with insulin. Participants in Dysch et al.'s (2012) study gave examples of boredom, depression or just not being in the mood to adhere to some of the demands of taking their medication or checking their blood glucose level. Conversely, if the person had a goal and *wanted* to self‐manage their diabetes this facilitated their ability to do so. In Whitehouse et al.'s (2016) research about negated autonomy in diabetes self‐management for people with intellectual disabilities, a participant had the goal to move out of a residential setting to live alone. She was being supported to learn how to manage her insulin injections to facilitate her independence.

#### 3.1.4 Self‐confidence

This contributed to one's ability to manage their diabetes with insulin within four of the studies. Hale et al. (2011) and Whitehead et al. (2016) found that a lack of self‐confidence was a barrier. For example, the woman who was motivated to learn how to inject her insulin independently so she could move into her own home lacked confidence and was working on this with her support worker. Having self‐confidence was also a facilitator in these studies, and in Cardol et al. (2012b) and Maine et al. (2017). Cardol et al. (2012b) explained that confidence in one's own abilities is necessary for using knowledge and developing the skills to self‐manage diabetes.

#### 3.1.5 Perceived negative aspects of needing insulin

Barriers to managing diabetes with insulin included fear/dislike of needles and injections (seven studies). Strategies to overcome this included support from the carers and district nurses (Paterson et al., 2020). (Dysch et al., 2012) had a participant who disliked needles but expressed being able to overcome this to tolerate her insulin injections. Needing insulin was associated with a negative impact on people's lives in six studies. Cardol et al.'s (2012b) participants expressed the inconvenience of it such as when one was asked for dinner ‘but I couldn't because I didn't have my insulin with me. That's a real pity’ (p. 354). In Hale et al.'s (2011) study, participants expressed finding it difficult to remember to regularly check blood glucose and feeling self‐conscious about doing it in public. Having to wait in for the nurse to come and support the person was also inconvenient. One participant expressed ‘I think it's kind of sucky…that you have to inject every day’ (p. 227). According to Paterson et al. (2020) the two most common reasons for not taking prescribed medication were forgetting to take it and side effects. Brown et al. (2017) highlighted that a simplified insulin management plan is sometimes implemented for people with intellectual disabilities, leading to higher blood glucose levels and increased diabetes complications risk.

However, requiring insulin was not always perceived as negative. A nurse in Cardol et al.'s (2012a) research expressed that they were more attentive towards a client who had diabetes and required insulin. Paterson et al. (2020) highlighted the importance of using insulin to optimise glycaemic levels and suggested that with support people with intellectual disabilities may reach comparable levels of medication‐taking to those without intellectual disability.

#### 3.1.6 Acceptance

Coming to terms with having diabetes and the need for treatment can facilitate diabetes management. Speaking of insulin, a participant in Dysch et al.'s (2012) study expressed ‘I know I've got to do it [insulin injections] to save my life’ (p. 43) ‘I'll just have to try and do my best with it’ (p. 44). Cardol et al. (2012b), Hale et al. (2011), Maine et al. (2017) and Whitehead et al. (2016) had participants who articulated that they were used to having diabetes and had adjusted to living with it. Accepting the need for support was also apparent. A participant from Whitehead et al.'s (2016) study expressed that ‘As much as possible I am managing my diabetes which is good. Sometimes I can't but I can get staff to help control it’ (p. 393).

#### 3.1.7 Symptom recognition (e.g., hypo awareness)

Being able to recognise the symptoms of high‐ or low‐blood glucose levels can support one's ability to manage diabetes with insulin. Dysch et al. (2012) found that participants engaged with their illness through describing the physical experience of living with diabetes. Participant Jane had symptoms if she omitted her medication, so this was a motivation to take it. Similarly, Hale et al. (2011) found that most participants were able to recognise when their blood glucose levels were incorrect. Maine et al. (2017) Found that symptom recognition was an important source of confidence in self‐management; having hypoglycaemia awareness enabled participants to act and gave them a sense of control over the physiological effects of living with diabetes. Being able to communicate symptoms to supporters was important, so that action could be taken if required (Whitehead et al., 2016).

#### 3.1.8 Physical disability

For some people with intellectual disabilities, physical disability made diabetes management with insulin more challenging according to three papers. Dysch et al.'s (2012) found that multiple health issues impacted on self‐management, such as visual impairment. This was also mentioned by Hale et al. (2011) and Maine et al. (2017). Strategies were identified to overcome difficulties, such as a large print glucose diary, a talking glucometer and support from a carer Dysch et al. (2012).

### 3.2 Theme 2: The role of other people

#### 3.2.1 Support

A strong theme throughout all the studies was the role of support from other people to facilitate diabetes management. The results indicate it is crucial that people with intellectual disabilities receive appropriate support from family, carers, support workers and healthcare professionals to optimise their ability to manage their diabetes with insulin. This included practical support to check blood glucose or inject insulin, manage diet and treat hypoglycaemia, and it also included encouragement and emotional support to facilitate autonomy and learn skills. There were differences in the level of support required and the approach towards it. For example, in Brown et al.'s (2017) study, diabetes healthcare professionals described high levels of support needs, whereas staff working within intellectual disability services identified higher levels of independence in some people with intellectual disabilities. Dysch et al. (2012) found that there was a struggle with the need for support because although participants in their study acknowledged that they needed help to manage their diabetes, it could be frustrating for them at times. Maine et al. (2017) found that autonomy for the person with diabetes was achieved through both the acceptance and rejection of support from others. Support was sometimes described as ‘doing’ in terms of physically injecting the insulin and checking the glucose (Whitehead et al., 2016) and other times it was about facilitating, encouraging, and enabling a person to be as independent as possible (Whitehead et al., 2016).

#### 3.2.2 Shared responsibility

Managing diabetes together and sharing responsibility was a facilitator according to all the results except for Maine et al. (2017). Cardol et al. (2012b) gave examples of people with intellectual disabilities who required support with their insulin administration, but were able to do some aspects themselves, such as doing the injection, or preparing equipment. Dysch et al. (2012) had a participant who required support staff to draw the insulin up for them, but they could inject the needle themselves. Whitehead et al. (2016) found that insulin administration and blood glucose testing was a negotiated process with support staff working together with people with intellectual disabilities as safely as possible to facilitate their autonomy.

#### 3.2.3 Attitudes

The attitudes of other people acted as a barrier or a facilitator to being able to manage diabetes with insulin. This was apparent from data from seven of the studies (Brown et al., 2017; Cardol et al., 2012a, 2012b, Maine et al., 2017, Rouse & Finlay, 2016 Trip et al., 2016 and Whitehead et al., 2016). Where there was a positive attitude from other people, this facilitated the person's self‐management abilities. In Whitehead et al.'s (2016) research support workers described their role as providing encouragement to support autonomy. Cardol et al. (2012a) gave examples of trust, positive, creative, and flexible approaches from caregivers to increase the person's confidence to self‐manage. Trip et al. (2016) found that support workers nurtured self‐management skills by enabling companionship with others, encouraging and supporting practical skills.

Negative attitudes were illustrated in Cardol et al.'s (2012a) study from caregivers, such as a social worker who spoke of a client with intellectual disability in relation to doing insulin injections: ‘I don't think he could do that himself, he lacks intellectual ability. I also think he would be too lazy’ (p. 385). This contrasted with a psychologist in Brown et al.'s (2017) study: ‘We need to increase people's autonomy, increase their informed choice… it's about empowering them’ (p. 443).

#### 3.2.4 Knowledge (access to education)

Diabetes knowledge and access to education/training for people who support those with intellectual disabilities and diabetes was a theme throughout seven papers. In Hale et al.'s (2011) research, participants with intellectual disabilities expressed that it was important to them that other people know how to support them, and in Whitehead et al.'s (2016) study there was evidence of how people with intellectual disabilities were actively involved in training their support workers to administer the insulin to them. However, this was not always positive: in Trip et al.'s (2016) study, four key workers stated that their knowledge of diabetes came solely from the person with intellectual disabilities themselves, highlighting a lack of formal training. All of the key workers in Trip et al.'s (2016) research wanted diabetes education and training to support those with diabetes. In Paterson et al.'s (2020) research into medication‐taking in people with intellectual disabilities, they found that a barrier to medication‐taking was side effects not being discussed with the person, with half of carers unaware of perceived side effects. The need for information for carers about side effects was advocated. Gregory (2019) emphasised the importance of training to ensure that community nursing staff are competent to deliver effective diabetes care, including insulin administration to people with intellectual disabilities. Participants in Brown et al.'s (2017) study recognised that diabetes healthcare professionals can benefit from education about intellectual disabilities specifically.

#### 3.2.5 Communication

The theme of communication emerged from all papers except Dysch et al. (2012) and Trip et al. (2016). This applied in relation to the person with intellectual disabilities and the people who support them, *and* regarding communication between supporters and healthcare professionals. Where there was effective communication, this facilitated diabetes management. For example, Maine et al. (2017) illustrated how positive communication from diabetes specialist nurses (DSNs) motivated a person with diabetes and they valued the encouragement from the DSN. Conversely, where communication was inadequate, people with intellectual disabilities had unanswered questions or lacked confidence to manage their diabetes. Cardol et al. (2012b) described a lack of communication between participants with intellectual disabilities and healthcare professionals. For example, although there was a link between knowledge and self‐management, one person on insulin expressed that they would not dare to ask their doctor questions. Rouse and Finlay (2016) described poor communication from doctors and the importance of having someone to support the person with intellectual disabilities in diabetes consultations.

Inadequate communication, presented as a barrier to diabetes management. Brown et al. (2017) explained how inaccurate communication leads to confusion and poor management and highlighted the need for improvements in communication and the development of networks between the different services that support people with intellectual disabilities and diabetes. This was regarded as part of delivering individualised and person‐centred care.

#### 3.2.6 Collaboration

Collaboration between people with diabetes, their supporters and healthcare professionals as a facilitator emerged from all papers excluding Dysch et al. (2012) and Cardol et al. (2012b). Brown et al. (2017) gave examples of intellectual disability practitioners collaborating with diabetes professionals to identify the most appropriate insulin and to work out how best to support people. Whitehead et al. (2016) described partnership and motivation between people with diabetes and their supporters as promoting autonomy. Rouse and Finlay (2016) found that responsibility for insulin was constructed as shared – staff worked together with the person with diabetes to make decisions and facilitate insulin administration. Brown et al. (2017) advocated closer collaborative working between diabetes and intellectual disability services to promote more effective practice and coordination of care.

#### 3.2.7 The needs of other service users

Barriers to supporting people with intellectual disabilities to manage their diabetes with insulin included the needs of other service users according to Brown et al. (2017), Cardol et al. (2012a) and Gregory (2019). This was primarily in relation to the provision of services and limited resources. For example, Gregory (2019) highlighted the increasing caseloads of people requiring support from community staff and the challenges in delivering this. However, Gregory's (2019) project aimed to improve service standards for people needing support (including those with intellectual disabilities who use insulin), illustrating that there are creative solutions to managing challenges.

#### 3.2.8 Conflict between protecting the person from harm versus facilitating their autonomy to self‐manage their diabetes

This was a theme within all papers except for Cardol et al. (2012b) and Gregory (2019). Rouse and Finlay (2016) found contradictory ideas regarding diabetes management in that people with intellectual disabilities should have independence and freedom of choice, but that those who have responsibility for supporting them should limit these choices to protect them from risky decisions. Similarly, Trip et al. (2016) found that key workers sometimes felt like they were ‘lifestyle police’ in terms of managing the person's diabetes with them. Hale et al. (2011) explained that staff supporting individuals with intellectual disabilities to manage their diabetes, found it difficult to know how much assistance to provide, especially if the person appeared knowledgeable and confident. Whitehead et al. (2016) found that support workers referred to the balance between benefit and risk; supporting people to stay healthy and maintaining autonomy. For example, a person with intellectual disabilities for whom the decision was made that he required someone trained in hypo/hyperglycaemia management to escort him when he goes out.

### 3.3 Theme 3: Social/environmental factors

#### 3.3.1 Reasonable adjustments and adaptations

The need for reasonable adjustments to facilitate diabetes management for people with intellectual disabilities who use insulin was a theme throughout the results, excluding Dysch et al. (2012). A flexible approach to care, resources and education that are adapted to the needs of people with learning disabilities and projects to improve support for people facilitated diabetes management (Brown et al., 2017; Gregory, 2019). For example, being accompanied to a diabetes consultation was very much appreciated by people with intellectual disabilities in Cardol et al.'s (2012b) study because health information could be explained to them later on. Conversely, if there were inflexible services, a lack of adapted resources such as structured education or inadequate access to EasyRead material, this was a barrier to optimal diabetes management (Brown et al., 2017).

#### 3.3.2 Person‐centred care

Person‐centred care was identified as a facilitator in five of the papers. This included the importance of seeing people as individuals and adapting to their requirements. For example, a psychologist in Brown et al.'s (2017) study expressed that people with intellectual disabilities and diabetes require tailored education and treatment packages to be fully informed and to achieve good health. However, time and resource limitations made it challenging to provide person‐centred care (Brown et al., 2017).

#### 3.3.3 Structure of services

The way services were structured had an influence on how people managed their diabetes with insulin (Brown et al., 2017; Gregory, 2019; Hale et al., 2011; Paterson et al., 2020; Trip et al., 2016). District nursing services regularly facilitate insulin administration but there are limitations (Brown et al., 2017) so an insulin regimen more than once a day can be challenging (even if it is appropriate). Furthermore, people with intellectual disabilities may find it restrictive having to wait at home for district nurses to come. In Whitehead et al.'s (2016) research, one participant did not want to be tied down to being at home for a second daily insulin visit from the nurse, although it may have improved her glycaemic levels. Gregory (2019) illustrated that there are creative ways of improving service standards for people who need support at home to manage their insulin, but her project required additional funding and as she points out, this can be challenging to sustain. Examples of positive working within services included multidisciplinary team working (Brown et al., 2017; Trip et al., 2016), and the professionals in Brown et al.'s (2017) study advocated a shared care pathway between the diabetes and intellectual disabilities teams to enhance care.

#### 3.3.4 Where the person lives

Having access to support from family, carers, peers or healthcare professionals may vary depending on where a person lives. For example, in supported living there may be a worker to support with insulin management, or friends to provide support and encouragement (Maine et al., 2017 and Whitehead et al., 2016). However, Maine et al. (2017) and Cardol et al. (2012b) found that the social setting was sometimes detrimental to self‐management, because of regimented routines, communal meals, and dietary temptations. For individuals living independently, some required daily visits from nurses to support with insulin administration (Hale et al., 2011). There were limitations on what support was available, or how frequently the nurses could visit (Brown et al., 2017; Gregory, 2019). This can result in compromises with insulin regimens leading to poorer glycaemic control and greater risk of long‐term complications (Brown et al., 2017).

##### The stigma of injecting insulin/checking blood glucose

This was a barrier to managing diabetes with insulin in Dysch et al. (2012) and Hale et al. (2011). For example, participants in Dysch et al.'s (2012) study expressed feeling uncomfortable or unable to inject in front of other people in social situations.

##### Technology

Technology to facilitate or inhibit diabetes management in people with intellectual disabilities using insulin, emerged briefly in the results. For example, issues with glucometers not working properly featured in Cardol et al. (2012a) and the benefits of a talking glucometer were mentioned by Maine et al. (2017). There were practical challenges to using an insulin pen described by Hale et al. (2011). In their discussion section, Hale et al. (2011) advocated for accessible websites for people with intellectual disabilities and the people who support them. Gregory (2019) was the only paper to mention flash glucose monitoring as a tool to facilitate diabetes management for people using insulin, as this is a relatively recent technological development.

## 4 DISCUSSION

In this review, 11 publications were identified to explore the barriers and facilitators to managing diabetes with insulin in people with intellectual disabilities. Diabetes management is determined by the complex set of interconnected factors related to the specific needs, preferences, and capabilities of individuals with intellectual disabilities and diabetes, attitudes of those supporting them in caring and clinical capacities, in the context of environmental/social determinants. Each factor acted as a barrier or a facilitator depending on the situation (illustrated in Tables 3, 4 and 5, Appendix S2). For example, the cognitive ability of the person was identified as a barrier to managing diabetes with insulin if the person was unable to understand and retain information about how to do it. However, it was also identified that if the person was perceived as competent and able, this facilitated diabetes management. To some extent, it depended on how *other* people perceived the person with intellectual disabilities to be able to manage their diabetes, and what support was available to facilitate this.

### 4.1 Supported self‐management

The concept of self‐management in diabetes is promoted among people with diabetes (Diabetes UK, 2009). Being unable to self‐manage can have negative connotations in terms of how the person perceives themselves, and how other people perceive them (Cardol et al., 2012a; Dysch et al., 2012). In reality, people with intellectual disabilities and diabetes are not the only ones in need of support, self‐management is complex and every person with diabetes requires support to some extent (Diabetes UK, 2009). For example, a relative assisting the person to inject their insulin or prepare their meals, a nurse advising insulin dose titration, or a healthcare professional reviewing the blood glucose results. Just like any other person on insulin, people with intellectual disabilities should be supported to manage their diabetes and empowered to self‐manage where appropriate. There is an expectation that this should be happening with ‘reasonable adjustments,’ and ‘supported self‐management’ should make it possible. However, as the reviewed evidence shows, people with intellectual disabilities face barriers.

### 4.2 Inequalities

There was evidence of inequalities. According to the Equality Act (2010), people with intellectual disabilities are entitled to reasonable adjustments to their care. Where there is a lack of reasonable adjustments to care, inflexible services, inadequate provision of diabetes education or negative attitudes from professionals (Brown et al., 2017; Cardol et al., 2012a), people with intellectual disabilities have more difficulty achieving optimal diabetes management with insulin. These issues go beyond diabetes care. The COVID‐19 pandemic has highlighted the stark inequalities facing people with intellectual disabilities. Williamson et al. (2021), revealed that people with intellectual disabilities have distinctly increased risks of hospital admission and death from COVID‐19. Courtney and Cooper (2021) stress that more work must be done to reduce the health inequalities exposed and amplified by the pandemic.

Despite barriers, attempts to reduce inequalities for people with intellectual disabilities who use insulin were evident throughout the data. There were positive attitudes expressed by some healthcare professionals and support workers who wanted to empower people with intellectual disabilities and facilitate their autonomy (Brown et al., 2017; Trip et al., 2016; Whitehead et al., 2016), and examples of reasonable adjustments such as extra appointment time and access to adapted resources. Gregory's (2019) service improvement project addressed some of the barriers for people who require support with their insulin, but such projects are not nationwide.

### 4.3 Education and training

The need for appropriate diabetes education for people with intellectual disabilities was apparent from this review. Participants with intellectual disabilities expressed wanting more knowledge and information about their diabetes (Cardol et al., 2012b; Hale et al., 2011). This is consistent with the findings of Maine et al. (2017), and Holden and Lee's (2021) systematic review about the barriers and enablers to optimal diabetes care for adults with intellectual disabilities. Holden and Lee (2021) found that current structured group education courses are not appropriate to the learning needs of adults with intellectual disabilities. Adapted programmes, such as DESMOND‐ID for people with type 2 diabetes and intellectual disabilities have been developed (Taggart et al., 2018), but such programmes are not available nationally (Maine et al., 2017). Accessible patient resources and information for people supporting those with intellectual disabilities are available (Diabetes UK, 2022), so it is important that people know how to access these.

Family, support staff and healthcare professionals require information, education, and training to be confident and competent to facilitate diabetes management with insulin for people with intellectual disabilities. Practical skills such as being able to use a glucometer or insulin injection are important, but this review demonstrates that knowing *how* to facilitate self‐management and be empowering towards people with disabilities are also essential to working in a person‐centred way. This is consistent with Maine et al.'s (2020) review into the experience of type 2 diabetes self‐management in adults with intellectual disabilities and their caregivers, which found overall inconsistency in staff knowledge and training. Maine et al. (2017) advocated caregiver training to support and enhance positive self‐perceptions in people with intellectual disabilities.

The passing of an amendment to the Health and Care Bill in England means that training in understanding the needs of people with intellectual disabilities for all NHS health and social care staff will be compulsory (Walker, 2022). This is a positive step, but gaps remain in specific training for people supporting individuals with intellectual disabilities to manage their diabetes.

### 4.4 Technology

Technology is increasingly important in diabetes management (Li & Hussain, 2020), yet there was little mention of this in the review data. This is likely to be because diabetes technology has accelerated in recent years. Gregory (2019) illustrated how flash glucose monitoring (where the person wears a sensor on their arm to monitor blood glucose) can facilitate diabetes management for people with intellectual disabilities who use insulin. According to new guidelines from the National Institute for Health and Care Excellence (NICE NG17, 2022), people with type 1 diabetes should be offered continuous glucose monitoring systems (CGMS) or flash glucose monitoring. People with type 2 diabetes, an intellectual disability and using insulin, should be offered a flash glucose monitor (NICE NG28, 2022).

None of the publications in this review referred to people with intellectual disabilities using insulin pump therapy. This suggests disparity in access to this technology, because according to NICE, adults with type 1 diabetes should be offered an insulin pump if the person cannot reach their target HbA1c without severe hypos, or if their HbA1c remains high despite carefully trying to manage their diabetes. Insulin pump therapy can be complex, requiring a high level of self‐management (Li & Hussain, 2020), but as technology evolves it may become more accessible to people with intellectual disabilities. There is a need for more research to explore how technology can facilitate diabetes management for people with intellectual disabilities.

## 5 LIMITATIONS OF THE DATA

The included publications were not research *specifically* focussing on diabetes management with insulin for people with intellectual disabilities, so a more in‐depth study to address this is required. There was a lack of patient and public involvement in the research processes of the papers, which needs to be addressed if research is to be collaborative, empowering, and high quality (NIHR, n.d.). In the studies which included people with intellectual disabilities as participants (Cardol et al., 2012b; Dysch et al., 2012; Hale et al., 2011; Maine et al., 2017; Paterson et al., 2020; Rouse & Finlay, 2016; Whitehead et al., 2016). The intellectual disability was described as ‘mild’ or ‘moderate’ – there was almost no reference to people with intellectual disabilities who are unable to participate in conventional verbal interviews or discussion about why they were excluded from participating. The needs of people with disabilities who cannot express themselves through interview have been largely ignored in the diabetes research to date, so inclusive research is recommended. Studies which include participant observation could enable exploration into people's lived experiences and healthcare practices.

### 5.1 Strengths and limitations of this review

This systemised review has offered insights into an issue that has previously been unexplored, and Patient and Public Involvement (PPI) was integral to the development of the research question. There was thorough analysis of each paper by both reviewers. However, there are limitations because the searches were limited to three databases and did not include grey literature, or papers not published in English. The authors do not have an intellectual disability or diabetes, and it is recognised that their interpretations may be different to those of people who have personal experience of these. PPI throughout the research process is proposed for future research.

## 6 CONCLUSION

Supported self‐management is crucial to optimising diabetes management and people with intellectual disabilities are entitled to reasonable adjustments to care, but this systemised review into the barriers and facilitators of managing diabetes with insulin for people with intellectual disabilities indicates that there are obstacles to achieving this. Person‐centred care, additional support, education, and training are required to appropriately address people's needs and to reduce inequalities. Gaps in the research have been identified, indicating the need for studies which are inclusive to people unable to participate in conventional verbal interview, and research which looks at the role of technology in supporting diabetes management. Studies designed with PPI are recommended to ensure that people with intellectual disabilities are at the heart of the research process.

## CONFLICT OF INTEREST

The authors declare no conflict of interest.

## Supporting information


**Appendix S1**: Supporting information.

## Data Availability

The data that support the findings of this study are available from the corresponding author upon reasonable request.
