# Supplementary material for: The barriers and facilitators to managing diabetes with insulin in adults with intellectual disabilities: A systemised review of the literature
Source: J Appl Res Intellect Disabil. 2022 Aug 18;35(6):1253–66. doi: 10.1111/jar.13027 (PMC9805117; doi:10.1111/jar.13027)
Supplement: Supplementary file 1 — Appendix S1: Supporting information. [file JAR-35-1253-s001.zip › JAR_13027_Appx1 Extraction Template &amp; CASP-SuppInfo.docx]

Appendix 1: Extraction template and adapted CASP checklist

Reviewer name:

Study:

| Study location  (Country, geographical location, study setting) |  |
| --- | --- |
| Aims and objectives |  |
| Population  (Who was included, were the views of people with intellectual disabilities included?) |  |
| Recruitment and sample  (Eligibility criteria, who was recruited, recruitment method, number of participants, attrition rate) |  |
| Methodological approach and methods  (Study design, data collection method, data analysis, participants characteristics) |  |
| Findings  (What was found) |  |
| Outcomes |  |
| Anything else of interest |  |
| Barriers to managing diabetes with insulin |  |
| Facilitators to managing diabetes with insulin |  |
| Relevant suggestions from authors |  |
| Quality   1. Was there a clear statement of the research aim? |  |
| 1. Was the methodology appropriate? |  |
| 1. Was the research design appropriate? |  |
| 1. Was there PPI in the research design? |  |
| 1. Was the recruitment strategy appropriate? |  |
| 1. Was data collected in an appropriate way? |  |
| 1. Were the perspectives of people with intellectual disabilities included? |  |
| 1. Was the relationship between researcher and participants adequately considered? |  |
| 1. Were ethical issues taken into consideration? |  |
| 1. Was data analysis rigorous? |  |
| 1. Was there a clear statement of findings? |  |
| 1. How valuable is the research |  |
| Overall quality: high/medium/low |  |
| Notes on inclusion/exclusion |  |
| Any other comments |  |

Adapted from Critical Appraisal Skills Programme (CASP, 2018)
