# Supplementary material for: The barriers and facilitators to managing diabetes with insulin in adults with intellectual disabilities: A systemised review of the literature
Source: J Appl Res Intellect Disabil. 2022 Aug 18;35(6):1253–66. doi: 10.1111/jar.13027 (PMC9805117; doi:10.1111/jar.13027)
Supplement: Supplementary file 1 — Appendix S1: Supporting information. [file JAR-35-1253-s001.zip › JAR_13027_Appx2 Tables 3 4 &amp; 5 Themes-SuppInfo.docx]

# The barriers and facilitators to managing diabetes with insulin in people with intellectual disabilities: themes

**Table 3. Theme: The individual**

|  | The individual |  |
| --- | --- | --- |
| Barriers | Subthemes | Facilitators |
| Unable (or perceived as unable by other people) to check blood glucose / inject insulin. | **Cognitive ability / Competence** | Able (or perceived as able by other people) to manage blood glucose testing / insulin injections. |
| - Lack of understanding - Lack of education about their diabetes. | **Knowledge (access to education)** | - Has understanding about their diabetes and why they need the insulin - Has access to information and education. |
| Low mood or not motivated to do own injections and/or check blood glucose levels. | **Motivation/mood** | Person is motivated to self-manage. May have a specific goal (e.g., to live independently). |
| Lacks confidence or is perceived as over-confident in self-management. | **Self-confidence** | Has confidence that they can do it; is supported to develop confidence. |
| - Inconvenience of checking blood glucose and having injections - Negative implications of hypoglycaemia - Fear of needles and injections. | **Perceived negative aspects of needing insulin** | - Able to overcome challenges with support - The person understands why they need the insulin and how to manage side effects. |
| Difficulty accepting the need for checking blood glucose and having injections. | **Acceptance** | Has come to terms with their condition and the need for treatment. |
| Hypoglycaemia unaware = needs support from others. | **Symptom recognition**  **(e.g., hypoglycaemia awareness)** | Has awareness of symptoms, so understands the need for treatment (hyperglycaemia = needs medication, hypoglycaemia = needs glucose). |
| Needs help to do blood glucose or insulin e.g., due to dexterity issues or visual impairment. | **Physical disability** | Strategies to overcome difficulties, such as large print glucose diary / talking glucometer, support from a carer. |

**Table 4. Theme: The role of other people**

|  | The role of other people |  |
| --- | --- | --- |
| Barriers | Subthemes | Facilitators |
| - Lack of support, inconsistences (e.g., due to staff turn-over) - The person with diabetes may sometimes struggle with the need for support. | **Support** | - Practical support e.g., to do blood glucose or inject insulin, manage diet, and treat hypos - Support to be autonomous and learn skills - Support from a range of sources: family, support workers, healthcare professionals, peers - Encouragement. |
| The challenge of knowing how much responsibility lies with the person themselves and how much lies with the people supporting them. | **Shared responsibility** | Working together to manage the diabetes. |
| Negative attitude to the person with diabetes, e.g., that they are lazy or cannot learn; inflexible approach to supporting them. | **Attitudes** | Positive attitudes of others to empower the person and facilitate their diabetes management. |
| Lack of training and understanding about diabetes and how to manage it. | **Knowledge (access to education)** | The person providing support needs knowledge and wants education/training about diabetes – may also need training about working with people with intellectual disabilities. |
| Inadequate/negative communication with the person or between those who support them. | **Communication** | - Communication between the person and the people who support them - Communication between supporters and healthcare professionals. |
| Lack of joined-up care; Staff not knowing about the person’s diabetes care plan. | **Collaboration** | Between the person with diabetes, their supporters, and healthcare professionals. |
| Other people also need support (limited resources). | **The needs of other service users** | Supporting each other. |
| Restricting the person: ‘lifestyle police’. | **Conflict between protecting the person from harm versus facilitating their autonomy to self-manage their diabetes.** | Person-centred approach to care; facilitating the person’s autonomy. |

**Table 5. Theme: Social/environmental factors**

|  | Social / environmental factors |  |
| --- | --- | --- |
| Barriers | Subthemes | Facilitators |
| - Lack of adapted resources such as structured education - Lack of / not accessing Easy Read material. | **Reasonable adjustments & adaptations** | - A flexible approach to care, resources and education that is adapted to the needs of people with intellectual disabilities - Projects to improve support for people. |
| - District nurse comes at set times for the injections - Lack of resources. | **Structure of services** | - Flexible. E.g., district nurse supporting with injections - Local projects to support people. |
| - No one to help with testing blood glucose / insulin - Regimented routines - Dietary temptations. | **Where the person lives** | Has access to support from family / carers / peers / healthcare professionals. |
| Person does not want to check blood glucose / inject insulin in front of others. | **Stigma of injecting/checking blood glucose levels** | Able to overcome this with support. |
| - Difficulty using glucometers / insulin device. - Not accessing flash glucose monitoring. | **Technology** | - Able to use glucometer / insulin pens - Access to flash glucose monitor. |
