# Supplementary material for: The barriers and facilitators to managing diabetes with insulin in adults with intellectual disabilities: A systemised review of the literature
Source: J Appl Res Intellect Disabil. 2022 Aug 18;35(6):1253–66. doi: 10.1111/jar.13027 (PMC9805117; doi:10.1111/jar.13027)
Supplement: Supplementary file 1 — Appendix S1: Supporting information. [file JAR-35-1253-s001.zip › JAR_13027_Appx3 Tables 67 &amp; 8 Results-SuppInfo.docx]

Appendix 3

Table 6. Results: Theme 1. The individual

| **Theme title→** | Cognitive ability / Competence | | Knowledge (access to education) | | Motivation/mood | | Self-confidence | | Perceived negative aspects of needing insulin | | Acceptance | | Symptom recognition | | Physical disability | |
| --- | --- | --- | --- | --- | --- | --- | --- | --- | --- | --- | --- | --- | --- | --- | --- | --- |
| **Study↓** | Barrier | Facilitator | Barrier | Facilitator | Barrier | Facilitator | Barrier | Facilitator | Barrier | Facilitator | Barrier | Facilitator | Barrier | Facilitator | Barrier | Facilitator |
| Brown et al (2017) High quality | ✓ |  | ✓ | ✓ |  |  |  |  |  |  |  |  |  |  |  |  |
| Dysch et al (2012) High Quality |  |  |  | ✓ | ✓ | ✓ |  |  | ✓ |  |  | ✓ |  | ✓ | ✓ |  |
| Hale et al (2011) High Quality | ✓ | ✓ | ✓ | ✓ |  | ✓ | ✓ | ✓ | ✓ |  |  | ✓ |  | ✓ | ✓ |  |
| Maine et al (2017) High Quality | ✓ | ✓ | ✓ | ✓ |  | ✓ |  | ✓ | ✓ |  |  | ✓ |  | ✓ | ✓ |  |
| Whitehead et al (2016) High Quality |  |  |  | ✓ |  | ✓ | ✓ | ✓ | ✓ |  |  | ✓ |  | ✓ |  |  |
| Cardol et al (2012a) Medium quality | ✓ |  | ✓ |  | ✓ |  |  |  |  |  |  |  | ✓ |  |  |  |
| Cardol et al (2012b) Medium quality | ✓ |  |  | ✓ | ✓ | ✓ |  | ✓ | ✓ |  |  | ✓ |  |  |  |  |
| Paterson et al (2020) Medium quality | ✓ |  |  |  |  | ✓ |  |  | ✓ | ✓ |  |  |  |  |  |  |
| Rouse & Finlay (2016) Medium quality | ✓ | ✓ |  | ✓ | ✓ |  |  |  | ✓ |  |  |  |  | ✓ |  |  |
| Trip et al (2016) Medium quality |  | ✓ |  | ✓ |  | ✓ |  |  | ✓ | ✓ |  |  |  | ✓ |  |  |
| Gregory (2019) Low quality | ✓ |  |  |  | ✓ |  |  |  |  |  |  |  | ✓ |  |  |  |

Table 7. Results: Theme 2. Other people (Family/carers/support workers/healthcare professionals)

| **Theme title→** | Support from other people | | Shared responsibility | | Attitudes of other people | | The knowledge of others and access to education for them | | Communication | | Collaboration | | The needs of other service users | | A conflict between protecting the person from harm versus facilitating their autonomy to self-manage their diabetes. |
| --- | --- | --- | --- | --- | --- | --- | --- | --- | --- | --- | --- | --- | --- | --- | --- |
| **Study↓** | Barrier | Facilitator | Barrier | Facilitator | Barrier | Facilitator | Barrier | Facilitator | Barrier | Facilitator | Barrier | Facilitator | Barrier | Facilitator |  |
| Brown et al (2017) High quality |  | ✓ |  | ✓ | ✓ | ✓ | ✓ | ✓ |  | ✓ |  | ✓ | ✓ |  | ✓ |
| Dysch et al (2012) High Quality | ✓ | ✓ | ✓ | ✓ |  |  |  |  |  |  |  |  |  |  | ✓ |
| Hale et al (2011) High Quality |  | ✓ |  | ✓ |  |  |  | ✓ | ✓ | ✓ |  | ✓ |  |  | ✓ |
| Maine et al (2017) High Quality | ✓ | ✓ |  |  | ✓ | ✓ |  |  | ✓ | ✓ |  | ✓ |  |  | ✓ |
| Whitehead et al (2016) High Quality |  | ✓ |  | ✓ |  | ✓ |  | ✓ |  | ✓ |  | ✓ |  |  | ✓ |
| Cardol et al (2012a) Medium quality |  | ✓ |  | ✓ | ✓ | ✓ | ✓ | ✓ |  | ✓ |  | ✓ | ✓ |  | ✓ |
| Cardol et al (2012b) Medium quality |  | ✓ |  | ✓ | ✓ |  |  |  |  | ✓ |  |  |  |  |  |
| Paterson et al (2020) Medium quality |  | ✓ |  | ✓ |  |  | ✓ |  |  | ✓ |  | ✓ |  |  | ✓ |
| Rouse & Finlay (2016) Medium quality |  | ✓ |  | ✓ | ✓ |  |  |  |  | ✓ |  | ✓ |  |  | ✓ |
| Trip et al (2016) Medium quality |  | ✓ |  | ✓ |  | ✓ | ✓ | ✓ |  |  |  | ✓ |  |  | ✓ |
| Gregory (2019) Low quality |  | ✓ |  | ✓ |  |  |  | ✓ | ✓ | ✓ |  | ✓ | ✓ |  |  |

Table 8. Results: Theme 3. Social/environmental factors

| **Theme title→** | Reasonable adjustments & adaptations | | Person-centred care | | The structure of services | | Where the person lives | | Stigma of injecting or checking blood glucose | | Technology | |
| --- | --- | --- | --- | --- | --- | --- | --- | --- | --- | --- | --- | --- |
| **Study↓** | Barrier | Facilitator | Barrier | Facilitator | Barrier | Facilitator | Barrier | Facilitator | Barrier | Facilitator | Barrier | Facilitator |
| Brown et al (2017) High quality | ✓ | ✓ |  | ✓ | ✓ | ✓ |  |  |  |  |  |  |
| Dysch et al (2012) High Quality |  |  |  |  |  |  |  |  | ✓ |  |  |  |
| Hale et al (2011) High Quality |  | ✓ |  |  | ✓ |  | ✓ |  | ✓ |  | ✓ |  |
| Maine et al (2017) High Quality |  | ✓ |  |  |  |  | ✓ | ✓ |  |  |  | ✓ |
| Whitehead et al (2016) High Quality |  | ✓ |  | ✓ |  |  |  | ✓ |  |  |  |  |
| Cardol et al (2012a) Medium quality |  | ✓ |  | ✓ |  |  | ✓ | ✓ |  |  | ✓ |  |
| Cardol et al (2012b) Medium quality |  | ✓ |  |  |  |  | ✓ | ✓ |  |  |  |  |
| Paterson et al (2020) Medium quality |  | ✓ |  |  |  | ✓ |  |  |  |  |  |  |
| Rouse & Finlay (2016) Medium quality |  | ✓ |  |  |  |  |  |  |  |  |  |  |
| Trip et al (2016) Medium quality |  | ✓ |  | ✓ |  | ✓ |  |  |  |  |  |  |
| Gregory (2019) Low quality |  | ✓ |  | ✓ | ✓ | ✓ |  | ✓ |  |  |  | ✓ |
